# Knock-Down of Cathepsin D Affects the Retinal Pigment Epithelium, Impairs Swim-Bladder Ontogenesis and Causes Premature Death in Zebrafish

**DOI:** 10.1371/journal.pone.0021908

**Published:** 2011-07-01

**Authors:** Carlo Follo, Matteo Ozzano, Vera Mugoni, Roberta Castino, Massimo Santoro, Ciro Isidoro

**Affiliations:** 1 Laboratorio di Patologia Molecolare del Dipartimento di Scienze Mediche and Centro di Biotecnologie per la Ricerca Medica Applicata dell'Università del Piemonte Orientale, Novara, Italy; 2 Molecular Biotechnology Center, University of Torino, Torino, Italy; Laboratoire Arago, France

## Abstract

The lysosomal aspartic protease Cathepsin D (CD) is ubiquitously expressed in eukaryotic organisms. CD activity is essential to accomplish the acid-dependent extensive or partial proteolysis of protein substrates within endosomal and lysosomal compartments therein delivered via endocytosis, phagocytosis or autophagocytosis. CD may also act at physiological pH on small-size substrates in the cytosol and in the extracellular milieu. Mouse and fruit fly CD knock-out models have highlighted the multi-pathophysiological roles of CD in tissue homeostasis and organ development. Here we report the first phenotypic description of the lack of CD expression during zebrafish (*Danio rerio*) development obtained by morpholino-mediated knock-down of CD mRNA. Since the un-fertilized eggs were shown to be supplied with maternal CD mRNA, only a morpholino targeting a sequence containing the starting ATG codon was effective. The main phenotypic alterations produced by CD knock-down in zebrafish were: 1. abnormal development of the eye and of retinal pigment epithelium; 2. absence of the swim-bladder; 3. skin hyper-pigmentation; 4. reduced growth and premature death. Rescue experiments confirmed the involvement of CD in the developmental processes leading to these phenotypic alterations. Our findings add to the list of CD functions in organ development and patho-physiology in vertebrates.

## Introduction

Cathepsin D (CD) is an aspartic protease resident in endosomal and lysosomal compartments of all eukaryotic cells [1], [2]. Within these acid compartments CD accomplishes the extensive or limited proteolysis of substrates (including pro-enzymes, pro-hormones and growth factors), performing a crucial role in tissue homeostasis. CD can also act on small substrates at physiological pH in the extracellular space and in the cytosol [3]–[5]. CD has been implicated in cell death [6], [7], extracellular matrix remodeling [8]–[10] and cancer development and metastasis [11]–[14]. Accumulating evidence point to a role of CD in various steps of development in vertebrates, from oocyte maturation to histogenesis, morphogenesis and remodeling of embryonic organs [15]–[18]. The hormonal regulation of CD expression in uterus and placenta suggests a possible role of this protease also in the growth of embryo and in the delivery in mammal species such as cat [19], bovine [20] and human [21]. Thus, any alterations of CD activity levels in these animals may cause adverse effects on reproduction.

The crucial importance of CD in organ development has been demonstrated in mouse and fruit fly knock-out models. CD^−^/^−^ mice do not manifest a pathologic phenotype at birth, suggesting that CD function is dispensable during embryonic development [22]. However, CD^−^/^−^ mice manifest abnormalities later in life and die in a state of anorexia before the 21st day post-natal [22], [23]. At two weeks CD^−^/^−^ mice exhibit weight loss in association with progressive atrophy of intestinal mucosa, followed by massive intestinal necrosis, disorganization of myocardiac fibers, thromboembolia and profound destruction of lymphoid cells in the spleen and thymus [22]. CD^−^/^−^ mice also show accumulation of autofluorescent ceroid lipofuscin (“aging pigment”) accompanied by neurodegeneration in retina and central nervous system [23]–[25] and, near the terminal stage, they develop seizures and progressive retinal atrophy, that eventually leads to blindness. Increased apoptosis observed in the thymus, thalamus and retina suggests that CD is essential for proteolysis of proteins regulating cell growth, and tissue homeostasis, remodeling and renewal [23].

CD^−^/^−^ *Drosophila melanogaster* also develops normally [26] and, in contrast to CD^−^/^−^ mice, is viable and fertile. However, like in CD^−^/^−^ mice, the lack of CD in flies causes progressive neuronal lipofuscinosis and neurodegeneration [26]. This same phenotype has been described in sheep [27], [28] and American bulldogs [29] carrying mutations that compromise CD activity.

Thus, loss of CD function negatively impacts on normal development and functioning of several tissues (epithelium, nervous and muscular) and organs (such as the brain, eye, myocardium and intestine). The prominent phenotype of CD knock-out would depend on the genetic background of the animal, that is on its ability to compensate for CD loss of function in a given tissue/organ.

The zebrafish (*Danio rerio*) is a useful research model in developmental biology and in pathology due to the similarity of its organs with humans' ones along with the availability of genomic data and the easy to introduce genetic manipulations [30]. A number of zebrafish mutants have been described that recapitulate the phenotype of human diseases such as DiGeorge syndrome [31], muscular dystrophy [32], vascular diseases [33], [34], liver disease [35] and hypo-pigmentation diseases [36], [37], among others.

In this study we analyze for the first time the phenotype of zebrafish depleted of CD by specific morpholino. Knock-Down (KD) of CD resulted in multi-systemic anomalies involving primarily the development of the swim-bladder, the retina epithelium and body length. Zebrafish devoid of CD activity showed a shortened lifespan. The active role of CD in these phenotypic alterations was confirmed in rescue experiments. The present data reveal a novel role of CD in the correct development and function of the retina in zebrafish, and possibly also in mammals.

## Results

### Cathepsin D mRNA is present in un-fertilized eggs and during the embryogenesis of zebrafish

Preliminary experiments performed with the splicing morpholino (S-MPO) resulted in the absence of obvious phenotypes. This was associated with the persistence of tiny amount of CD in zebrafish larvae at 4 day post-fertilization (dpf). Several maternal mRNAs and proteins, among which cathepsin S and nothepsin, have been found in the zebrafish oocyte [38]. We therefore considered the possibility that CD mRNA and/or protein could be present in un-fertilized eggs (UFE).

**Figure 1:**
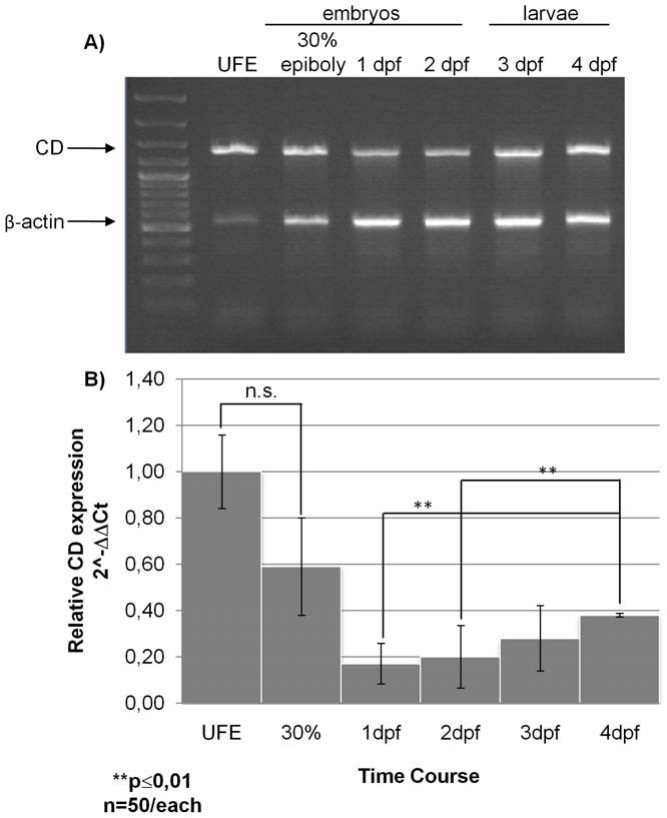
Zebrafish cathepsin D mRNA expression. **A**) Agarose gel electrophoresis of RT-PCR products. Total RNA was extracted from un-fertilized eggs (UFE), embryos at 30% epiboly, 1 dpf and 2 dpf, and larvae at 3 dpf and 4 dpf. Multiplex RT-PCR was performed using both zebrafish CD and β-actin 1 cDNAs specific primers into the same reaction. The products of the expected size are indicated by the arrows (the 549 bp amplicon for β-actin 1 and the 1380 bp amplicon for CD). DNA ladder was run in lane 1. Data reproduced in two other independent experiments. **B**) Relative transcript expression of zebrafish CD as assessed by qReal-Time PCR in UFE, embryos 30% epiboly, 1 dpf, 2 dpf and larvae 3 dpf and 4 dpf. Data and relative statistics (**, p<0.01; n.s., not significant) from two independent experiments in triplicate.

We first checked for the presence of maternal CD mRNA in wild type UFE by RT-PCR. We performed a multiplex RT-PCR for both CD and β-actin 1 mRNAs in UFE and in cells of zebrafish at different stages of development starting from 30% epiboly (corresponding to approximately 4.7 h post-fertilization, hpf) to the larva stage at 4 dpf [39]. This experiment demonstrated that CD mRNA is present in UFE and its expression is maintained at high basal level throughout the considered developmental stages (Fig. 1A). Since only one isoform of CD mRNA was detected using primers designed against its 5′/3′-UTRs sequence, we may conclude that no alternative splicing occurs. To better assess the level of CD mRNA expression during embryogenesis and development of zebrafish we performed a quantitative Real Time PCR (qReal-Time PCR) using β-actin as reference gene. Based on 2∼∧ΔΔCt data, the expression of CD mRNA increased with time, and by 4 dpf it reached a value two-fold that measured at 1 or 2 dpf (Fig. 1B). This increase was statistically highly significant (p<0.01). Apparently, the expression of CD mRNA decreases from UFE to 30% epiboly stage and to 1 dpf. This drop likely reflects the combined effect of the decay of the maternal CD mRNA and of the concomitant increase of actin mRNA (see also Fig. 1A).

### Mature cathepsin D is present during zebrafish development

**Figure 2:**
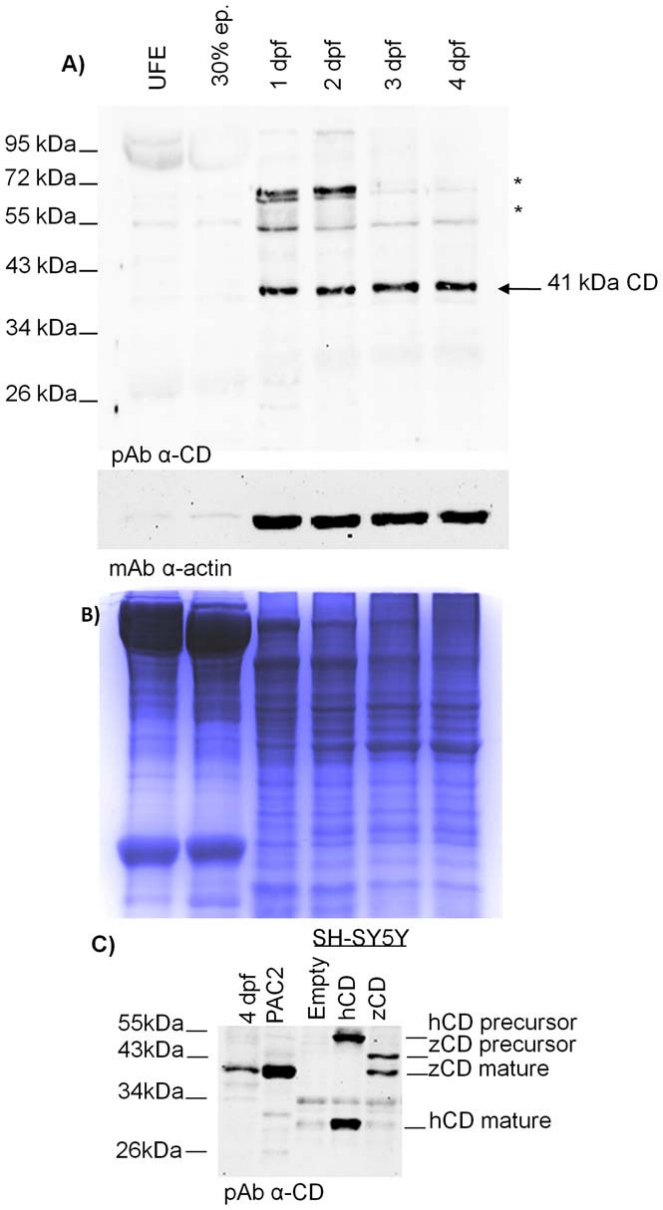
Zebrafish cathepsin D protein expression. **A**) Western blotting of CD in homogenates of un-fertilized eggs (UFE), embryos (at 30% epiboly, 1 dpf and 2 dpf) and larvae (at 3 dpf and 4 dpf). The filter was first probed with a polyclonal antibody against rat CD, stripped and re-probed with a monoclonal antibody against β-actin. The arrow points to the single-chain CD (41 kDa). Asterisks point to aspecific bands. The position of standard molecular weight proteins is indicated. One representative gel out of five independent experiment is shown. **B**) Colloidal Coomassie G-250 stained gel (after blotting) showing whole homogenate proteins of each sample loaded. **C**) Western blotting validation of the polyclonal anti-CD antibody against homogenates of 4 dpf larvae, PAC2 cells and SH-SY5Y cells (transfected with either the empty vector or the vector carrying the cDNA for human CD or for zebrafish CD). The position of the CD molecular forms recognized by the antibody is indicated on the right. The position of standard molecular weight is indicated on the left. Essentially a similar pattern of CD expression was obtained in two other independent experiments.

We have cloned and sequenced the CD cDNA of zebrafish generated by RT-PCR from 4 dpf larvae. Sequencing analysis indicated that CD mRNA codifies for a 41 kDa single-chain protein which is mono-glycosylated (data not shown). We checked whether CD protein is present in the egg of zebrafish before its fertilization (e.g., included by endocytosis during oocyte maturation). Wild type UFE, embryos (at 30% epiboly, 1 and 2 dpf) and larvae (at 3 and 4 dpf) were collected and analyzed for CD expression. Immunoblotting was performed with a rabbit polyclonal antiserum raised against rat CD in our laboratory [40], [41]. The ability of this antibody to specifically recognize zebrafish CD was ascertained in separate experiments (see below). The polyclonal antibody detected a main band of 41 kDa molecular weight, corresponding to the single-chain mature CD (Fig. 2A, upper panel, arrow), starting from 1 dpf embryo. The level of CD protein, normalized to actin, slightly increased with time of development, in agreement with mRNA data. No detectable CD protein is present before the fertilization and at 30% epiboly stage, despite the presence of its mRNA. Given that synthesis and maturation of CD requires >1.5 h [42], the above finding could be explained assuming that CD is not synthesized in UFE, while at 30% epiboly (4.66 hpf) the amount of CD protein accumulated is still under the detection limit of the western blotting. Coomassie blue staining of the protein loaded on gel shows a prominent band at high molecular weight in the homogenates of UFE and 30% epiboly (Fig. 2A, lower panel). This band likely contains vitellogenin, since it is missing in samples from 1 to 4 dpf that had been de-yolked prior to homogenization.

The polyclonal antibody produced in our laboratory specifically recognizes rodent and human CD [43]. We tested its ability to also recognize zebrafish CD in transfected human neuroblastoma SH-SY5Y cells over-expressing transgenic zebrafish CD. To this end, we performed a western blotting experiment in which the homogenates of transfected SH-SY5Y cells over-expressing human CD, of zebrafish embryonic fibroblast PAC2 cells and of 4 dpf larvae were also included as controls. In accord with our previous report [43], the antibody recognized the human CD forms (the precursor and the mature large chain) of expected size (Fig. 2C). The antibody also detected a band of 41 kDa, that corresponds to the predicted size of mature zebrafish CD, in homogenates of zebrafish larva, of PAC2 cells and of zebrafish cDNA-transfected SH-SY5Y cells. In the latter sample, a band of 43 kDa, that corresponds to the expected size of zebrafish pro-CD, was also detected (Fig. 2C). It is to note that the precursor of CD (either human and zebrafish) is detectable only in transfected over-expressing cells. Also, it is remarkable that contrary to human CD, that completes its maturation becoming a double-chain, zebrafish CD remains (mainly) as a single-chain protein. This is a common feature of fish CD [44], [45].

**Figure 3:**
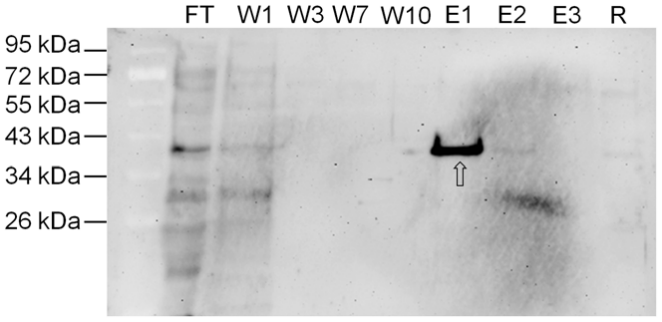
Zebrafish cathepsin D purification by affinity chromatography. Western blotting of CD purified from 4 dpf zebrafish homogenate after purification by pepstatinyl-agarose affinity chromatography. FT =  flow-through; W1-3-7-10 =  washing fractions; E1-2-3 =  eluted fractions; R =  fraction of residual proteins after elution. Fractions were desalted, concentrated, denatured in Laemmli buffer, resolved by SDS-PAGE and blotted onto nitrocellulose. The filter was incubated with polyclonal antibody against rat CD. The position of standard molecular weight proteins is indicated. The arrow points to 41 kDa zebrafish CD.

To further confirm the predicted molecular weight of mature zebrafish CD, we performed a purification of its active isoform by affinity chromatography using a pepstatinyl-agarose column (Fig. 3). The binding of CD to Pepstatin A occurs through the active site [46] and strictly depends on the proper folding of the mature polypeptide [41], [47]. The purified fractions were resolved by SDS-PAGE and the proteins transferred onto nitrocellulose filter were revealed with our rabbit polyclonal anti-CD antiserum [40], [41]. The experiment confirms that the molecular weight of the mature and enzymatically active zebrafish CD is of 41 kDa, as predicted by its cDNA, and that the protease exists as a single-chain isoform, in agreement with the above data. This molecular form of zebrafish CD was shown active toward several substrates at acid pH and in a Pepstatin A-sensitive manner, as revealed by enzyme assay using PAC2 cell homogenate as source (data not shown).

Taken together, the above data support the view that enzymatically active CD is present during zebrafish development, implying a potential role for this protease in this process.

### Assessment of Cathepsin D knock-down by two different morpholino oligonucleotides in Zebrafish

**Figure 4:**
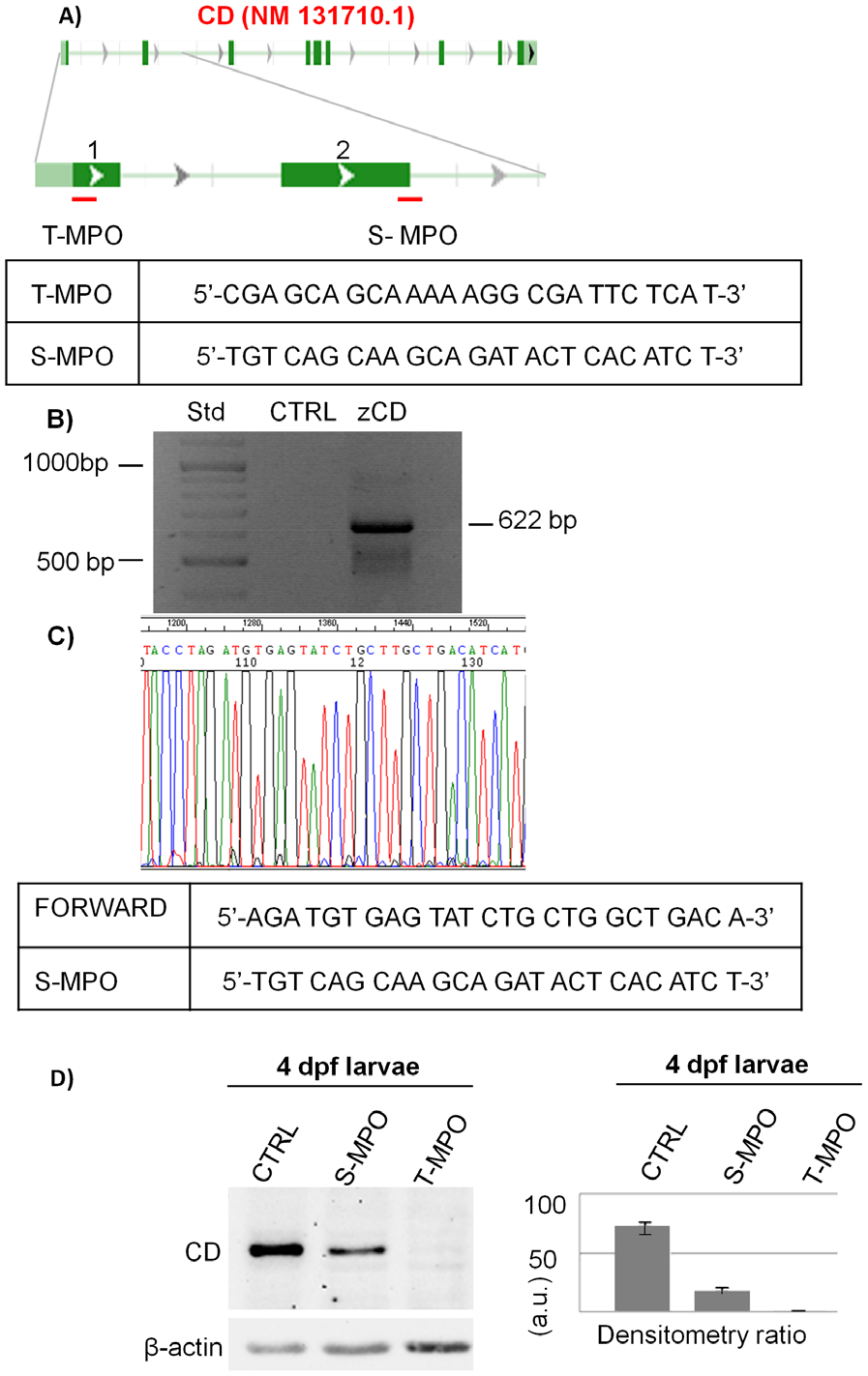
Zebrafish cathepsin D down-regulation by two different morpholinos. **A**) Schematic representation of S-MPO and T-MPO target regions in the CD RNA. The sequences of both MPOs are shown. **B**) Cloning of the 622bp, comprehensive of the S-MPO complementary site, from the genomic DNA of 4dpf larvae. **C**) Portion of the sequence of the 622bp region corresponding to the S-MPO complementary site. This experiment was performed two times with identical results. D) Western blotting of CD in homogenates of 4 dpf larvae deriving from control injection (CTRL), injection with splicing morpholino (S-MPO) and injection with translation morpholino (T-MPO). The filter was incubated with polyclonal antibody against rat CD, stripped and re-probed with monoclonal antibody against β-actin. The densitometry ratio of CD *vs* actin expression calculated from 5 independent experiments in double is shown. (a.u. =  arbitrary units).

Mature CD was not found in UFE and 30% epiboly embryos. Still, the mRNA analysis suggested the presence of maternal CD mRNA that could drive the synthesis of CD in post-fertilization stages. To achieve the extensive down-regulation of CD protein expression in fertilized eggs, we designed two different morpholino oligonucleotides targeting two different sites of the CD mRNA, so that both splicing and translation events could be disrupted (Fig. 4A). The S-MPO (splicing morpholino) was aimed at impairing the exon 2-intron 2 splicing in newly synthesized CD RNA, while the T-MPO (translation morpholino) was designed to affect the translation process of both mature maternal (pre-existing) and immature neo-synthesized CD RNAs. T-MPO, in fact, targets a region containing the ATG starting codon. To verify the specificity of S-MPO, we cloned and sequenced from genomic DNA a region of 622 bp (Fig. 4B) that includes the complementary site of S-MPO. Based on sequencing data (Fig. 4C) we can exclude the presence of polymorphisms that could hamper the ability of S-MPO to specifically match with its target in this region. We micro-injected wild type zebrafish fertilized eggs at the one/two-cell stage with standard control morpholino oligonucleotide (control injection, CTRL), S-MPO, or T-MPO. Micro-injections with either control MPO or the micro-injection solution alone generated larvae with identical wild type phenotype (not shown).

To test the efficacy of this KD approach, we assayed by western blotting the expression of mature CD at 4 dpf stage of development, that is at the larva stage in which CD expression (apparently) reaches the highest level (see above). Data shown in Fig. 4D demonstrate that in 4 dpf larvae the S-MPO injection reduced by approximately 5-fold the expression of CD, while the T-MPO injection achieved a complete KD of CD expression. Thus, in S-MPO larvae a residual 20% of CD persisted, likely arising from translation of maternal mRNA.

### Developmental effects of cathepsin D knock-down in Zebrafish

**Figure 5:**
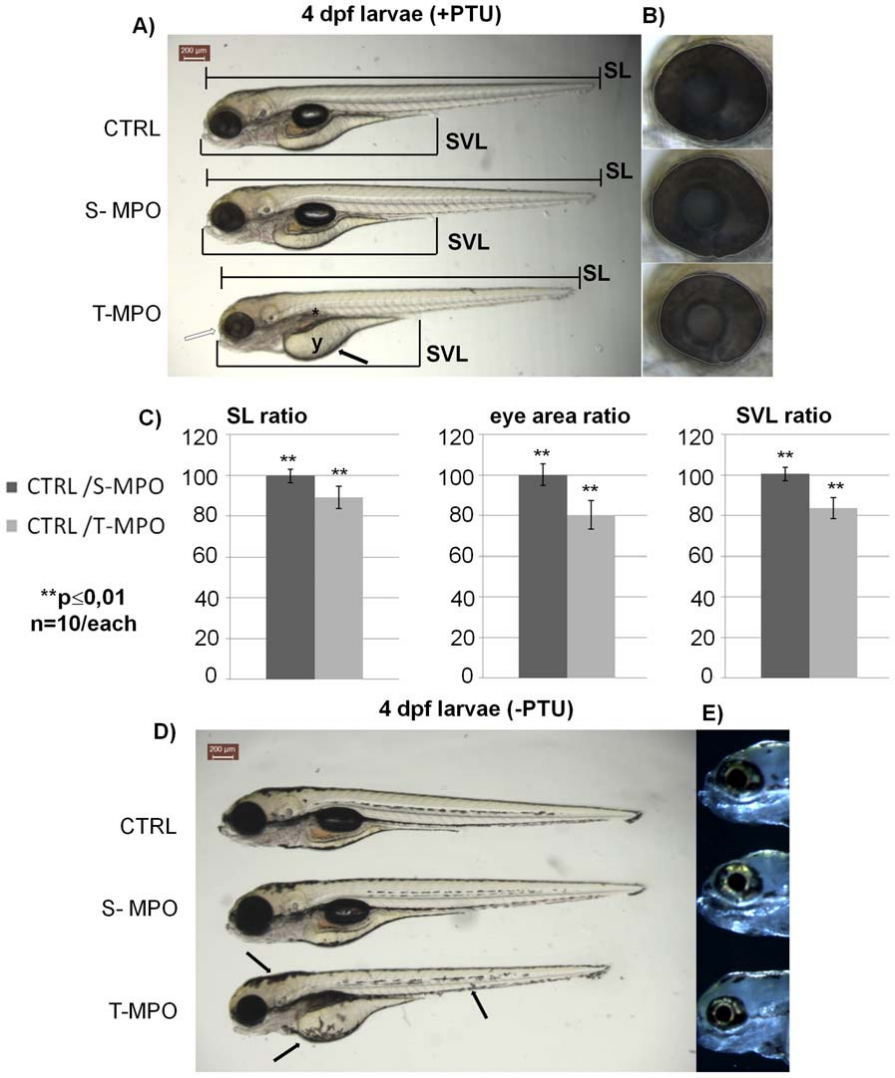
Phenotype of zebrafish cathepsin D following down-regulation by two different morpholinos at 4 dpf. Representative images of 4 dpf larvae resulting from control injection (CTRL), injection with S-MPO or with T-MPO. **A**) Larvae grown in the presence of PTU (+PTU). The main phenotypic alterations produced by T-MPO CD KD were: 1. microphtalmia (empty arrow); 2. absence of inflated swim-bladder (asterisk); 3. reduced yolk adsorption (arrow); 4. reduced body length (standard length, SL); 5. reduced oro-anal tract length (snout-vent length, SVL). **B**) Eyes magnification of 4 dpf larvae grown in the presence of PTU; the eye area is enclosed by the dotted circle. **C**) Body length, oro-anal tract length and eye area ratios calculated between CTRL and S-MPO or CTRL and T-MPO larvae. Measurements were done with the ImageJ software. Ten image sets obtained from 5 different experiments were analysed. Data are given as mean ± S.D. Differences in SL, SVL and eye area data were statistically highly significant (**, p ≤0,01) according to Student's *t* test. **D**) Larvae grown in the absence of PTU (-PTU). Following T-MPO CD knock-down the larvae showed skin hyper-pigmentation (arrows). **E**) Eyes magnification of 4 dpf larvae grown in the absence of PTU: dark-field images show normal iridophore reflections. Scale bar in **A** and **D** is 200 µm. Data presented in this figure have been reproduced in five independent experiments.

Next, we investigated whether the expression of the 41 kDa single-chain CD was indeed functional for the development of zebrafish. We analyzed the effects of CD down-regulation by comparing the gross phenotypic alterations produced by the two morpholinos at 4 dpf larva stage. Zebrafish were grown in the absence or the presence of the melanin-synthesis inhibitor PTU, so that pigmented and completely translucent larvae could be studied. Control MPO-injected fish developed normally and met the predicted developmental milestones [48]. External analyses of S-MPO- and T-MPO-larvae revealed that the former were practically indistinguishable from controls, while the latter presented several phenotypic alterations (Fig. 5A). T-MPO 4 dpf larvae presented the following phenotype: 1. reduction of the whole body length (standard length, SL) and of the oro-anal segment (snout-vent length, SVL) (Fig. 5A); 2. skin hyper-pigmentation with melanophores dispersed over the yolk (Fig. 5D); 3. absence of inflated swim-bladder (Fig. 5A,D); 4. impairment of yolk absorption (Fig. 5A,D); 5. microphtalmia (Fig. 5B,E). We also observed a reduced dimension of the posterior otolith of inner ear and of the semicircular canals (Fig. 5A,D). However, this phenotype was not evident in a portion of T-MPO larvae and therefore was not further investigated in the present work. SL and SVL in T-MPO larvae were 10% and 18%, respectively, shorter than in paired controls (Fig. 5C). In addition, the whole area of T-MPO larvae eyes was some 20% smaller than that of controls (Fig. 5C). It is to be noted that (apparently) the iridophore was not affected by the lack of CD during development (Fig. 5E). The lack of CD activity has been shown to be causally associated with neuronal lipofuscinosis [24], [26]–[29]. However, preliminary observations did not reveal such a phenotype in zebrafish larvae at 4 dpf, probably because of the relative short time.

The absence of a phenotype in S-MPO zebrafish could be explained by the presence of maternally supplied mature mRNA which guaranteed a sufficient amount of CD protein in the initial stages of embryo development.

### Rescue of T-MPO phenotype by mutated zebrafish CD mRNA

**Figure 6:**
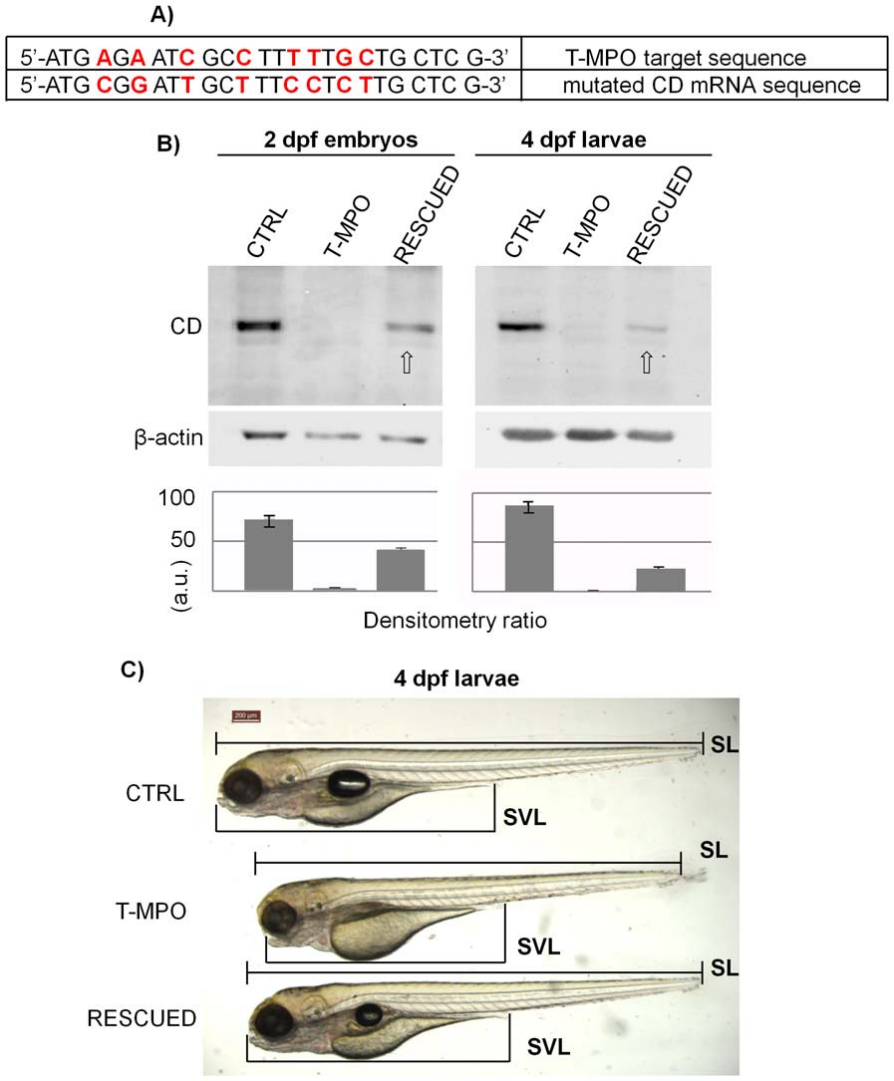
Rescue of T-MPO phenotypes by mutated zebrafish CD mRNA. **A**) T-MPO target sequence and corresponding mutated sequence of the *in vitro* synthesized CD mRNA. **B**) Western blotting of CD in homogenates of 2 dpf embryos and 4 dpf larvae deriving from control injection (CTRL), injection with T-MPO alone (T-MPO) and injection with T-MPO plus 200 pg/egg of mutant CD mRNA (RESCUED). The filter was incubated with polyclonal antibody against rat CD, stripped and re-probed with monoclonal antibody against β-actin. The densitometry ratio of CD *vs* actin expression is shown. (a.u. =  arbitrary units). Data reproduced in two to three independent experiments. The empty arrow points to CD expressed by the exogenous mRNA. **C**) Representative image of 4 dpf larvae obtained from control injection (CTRL), injection with T-MPO alone or with T-MPO plus 200 pg/egg of mutant zebrafish CD mRNA (RESCUED). Larvae body length (SL) and oro-anal tract length (SVL) are indicated. The experiment shows that synthesis of protein CD driven by exogenous CD mRNA in T-MPO injected zebrafish was sufficient to rescue a normal phenotype. Scale bar in **C** is 200 µm. Data presented in this figure have been reproduced in three independent experiments.

To definitively demonstrate that the lack of CD underlies all the phenotypic alterations observed in T-MPO zebrafish, a rescue experiment was performed. For this purpose we used an *in vitro* synthesized CD mRNA carrying eight nucleotide mutations in the matching sequence targeted by the morpholino (Fig. 6A). We micro-injected wild type zebrafish fertilized eggs at the one/two-cell stage with standard control morpholino oligonucleotide (control, CTRL), T-MPO, or T-MPO along with the mutant CD mRNA (RESCUED). We have assessed the optimal amount of exogenous mRNA needed to restore CD expression in the rescue experiments. In total we analyzed n = 40 embryos at 2 dpf (from two independent experiments) and n = 60 larvae at 4 dpf (from three independent experiments) for each condition. As shown by western blotting analysis (Fig. 6B), the mutant CD mRNA escaped the targeting by T-MPO and could be efficiently translated. The accumulation level of the CD protein in RESCUED zebrafish decreased with time according to the physiological decay of mRNA, which indirectly prove that its synthesis was indeed driven by the exogenous mRNA (Fig. 6B). External examination of the larvae at 4 dpf shows that in the rescued zebrafish the swim bladder formed and inflated normally, the yolk was adsorbed and the whole eye area and length of the body (SL) and of the oro-anal segment (SVL) were comparable with those of controls (Fig. 6C). Thus, the amount of newly-synthesized CD coded by the rescuing mRNA in the initial stages was sufficient to prevent the developmental anomalies imparted by T-MPO.

### Cathepsin D plays a key role in development of the retinal pigmented epithelium in zebrafish

**Figure 7:**
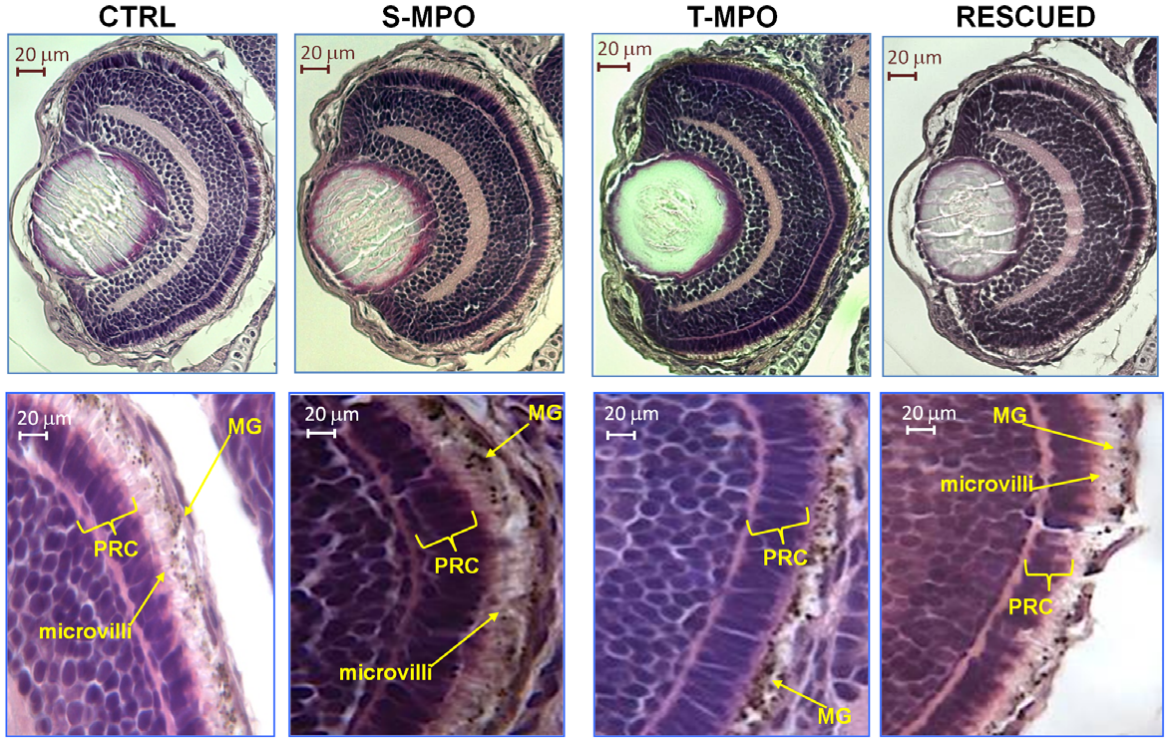
Role of cathepsin D in eye development. Hematoxylin-eosin staining of eye sections derived from 4 dpf micro-injected larvae (CTRL =  control injections; S-MPO =  splicing morpholino; T-MPO =  translation morpholino; RESCUED =  translation morpholino plus 200 pg/egg of mutant CD mRNA). Images at high magnification clearly show the palisade of microvilli of RPE cells (which contain melanin granules, MG) that interdigitate in the layer of photoreceptor cells (PRC) in CTRL, S-MPO and RESCUED zebrafish, while this is completely absent in T-MPO CD KD zebrafish (note the RPE cells containing melanine granules close to the PRC layer). Scale bar is 20 µm. Images representative of five (three for Rescued) independent experiments.

**Figure 8:**
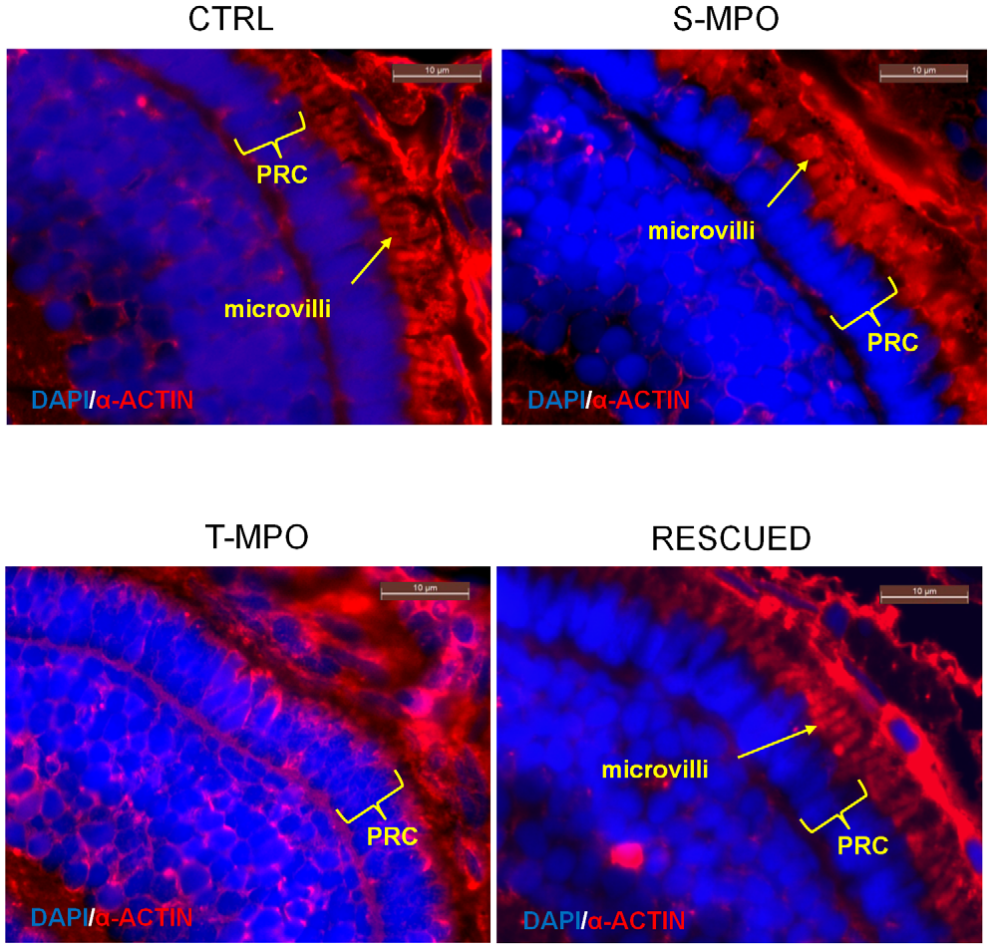
Immunofluorescence staining of microvilli in the eye of zebrafish following cathepsin D knock-down and rescue. Immunofluorescence staining of α-actin (red) in eye sections derived from 4 dpf micro-injected larvae (CTRL =  control injections; S-MPO =  splicing morpholino; T-MPO =  translation morpholino; RESCUED =  translation morpholino plus 200 pg/egg of mutant CD mRNA). Nuclei are stained with DAPI (blue). The arrow points to microvilli of RPE cells. Note the absence of this structure in T-MPO zenbrafish. The photoreceptor cell (PRC) layer is indicated by curly brackets. Scale bar is 10 µm. Images representative of five (three for Rescued) independent experiments.

**Figure 9:**
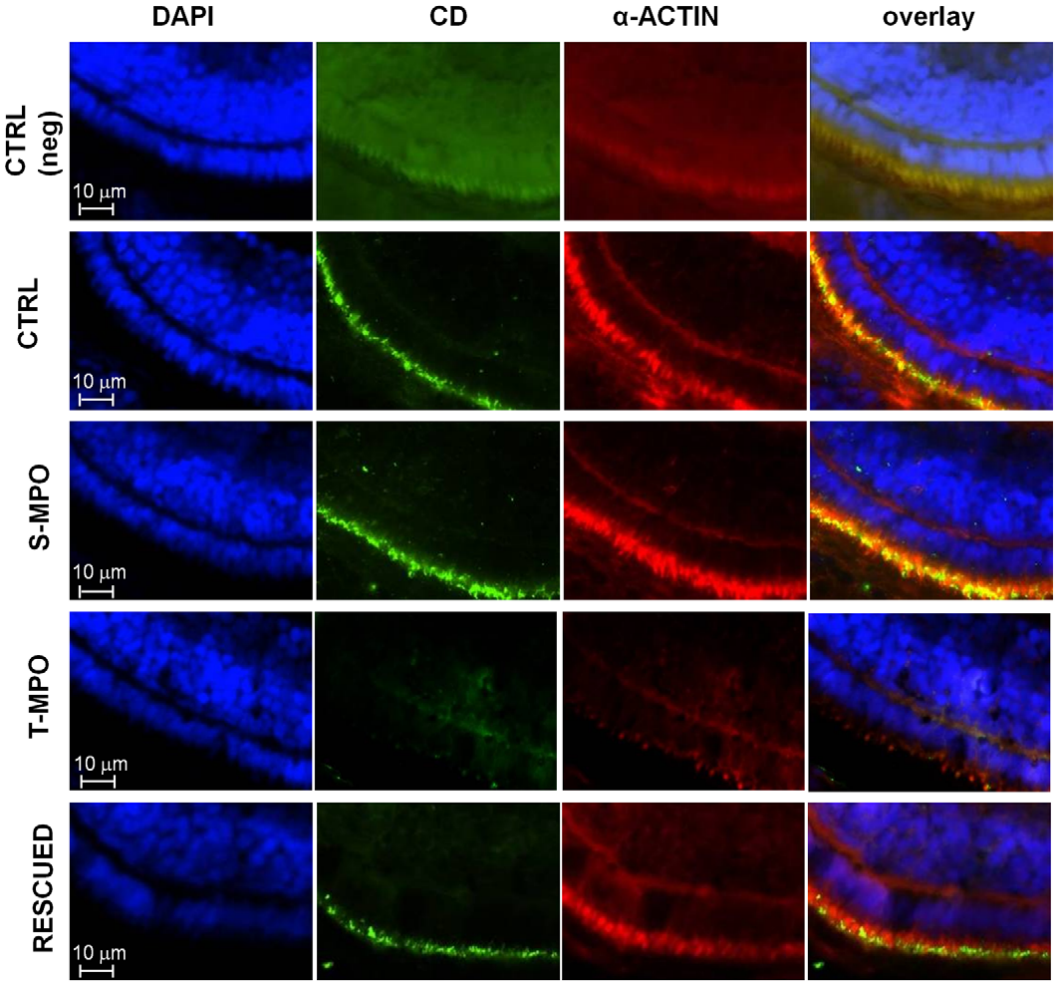
Immunofluorescence staining of CD in microvilli of RPE cells in zebrafish following cathepsin D knock-down and rescue. Immunofluorescence staining of CD and α-actin in eye sections derived from 4 dpf micro-injected larvae (CTRL =  control injections; S-MPO =  splicing morpholino; T-MPO =  translation morpholino; RESCUED =  translation morpholino plus 200 pg/egg of mutant CD mRNA). Nuclei are stained with DAPI (blue). As negative control, CTRL larvae sections have been incubated only with secondary antibodies (neg). Note the intense staining for CD in the RPE layer (identifiable by the actin-positive microvilli) in CTRL, S-MPO and RESCUED zebrafish. This experiment confirms the efficient KD of CD by T-MPO in agreement with western blotting data. These images are representative of three independent experiments. Scale bar is 10 µm.

Microphtalmia associated with defective pigmentation of the retinal pigmented epithelium (RPE) has been reported in zebrafish bearing genetic defects that affect the formation and traffic of lysosomal organelles [49] or their internal pH [37]. We therefore investigated more in detail at hystological level the eye damage caused by the lack of CD in T-MPO morphants. Microscopic analysis of eye transversal sections stained by hematoxylin-eosin showed an altered development of the retina in T-MPO morphants (Fig. 7). In particular, the layer of RPE showed reduced thickness and cellular disorganization. Most importantly, RPE cells in T-MPO morphants were avoided of the microvilli that normally interdigitate with photoreceptor cells (PRC). It is to be noted that the microvilli palisade in S-MPO and in rescued morphants is present and its thickness and organization do not differ from controls (Fig. 7). Microvilli are mainly composed by actin. Immunofluorescence staining of transversal eye sections with monoclonal anti-actin antibody allowed a better analysis of this phenotype. The images shown in Fig. 8 confirm the altered development of the RPE layer caused by the lack of CD in T-MPO zebrafish eye and the rescue of this layer in the eye of larvae that had been co-injected with T-MPO and mis-match CD mRNA. To definitively involve CD in this altered phenotype, we performed a double immunostaining of CD and α-actin in the RPE layer. Counter staining with DAPI evidenced nuclei position. The images in Fig. 9 demonstrate the absence of CD in RPE cells from T-MPO larvae and the presence of CD in RPE cells from S-MPO and RESCUED larvae as in controls.

## Discussion

The role of CD in organism development has been assessed in mice and insects [22]–[26]. In humans no phenotype of CD homozygous deletion has been described, very likely because such a condition would be lethal in the very early stages of embryogenesis. Still, it cannot be excluded that localized somatic mutation or epigenetic modulation affecting CD activity in restricted areas could result in altered development and homeostasis of confined tissue/organs. The present study provides the first phenotypic description of the lack of the lysosomal aspartic protease CD during zebrafish development. The data here reported demonstrate that zebrafish expresses only one isoform of CD mRNA (of the expected size 1380 bp) that drives the synthesis of a pro-CD of 43 kDa which is then converted into a mature, enzymatically active, single-chain protein of 41 kDa. The role of CD in zebrafish development was determined by morpholino-mediated KD and was definitively confirmed by rescue experiments. We observed a phenotype of zebrafish CD KD, showing multi-systemic anomalies, only when CD mRNA was targeted by a morpholino (T-MPO) interfering with the translation process. S-MPO, which targeted a splicing sequence in newly synthesized CD pre-mRNA in fertilized eggs, greatly, yet not completely, down-regulated CD expression. The phenotype of S-MPO larvae was indistinguishable from wild type. These observations indicate that: 1. maternal mature CD mRNA pre-exists in UFE and effectively drives the synthesis of CD upon fertilization, and 2. the translation of this mRNA provides a sufficient amount of CD to guarantee the (apparent) normal development of the embryo. At 4 dpf T-MPO-mediated CD KD zebrafish larvae presented with several phenotypic alterations, including failure of yolk absorption, reduced growth of the whole body and of the digestive tract, the lack of the swim bladder, microphtalmia and the disorganization of the RPE. All these alterations could be attributed uniquely to the lack of CD, based on the fact that rescuing CD protein synthesis by co-injecting a non-T-MPO sensitive mutant CD mRNA along with T-MPO led to the complete rescue of the normal phenotype, including the correct organization of the RPE layer. ‘Rescued’ larvae at 4 dpf expressed approximately 20% of the CD protein present in controls, an amount comparable with that found in S-MPO 4 dpf larvae. Thus, a relatively small amount of active CD in the very early stage of development is sufficient to allow the normal growth of zebrafish embryo. This strengthens the importance of CD-mediated proteolysis in embryo-morphogenesis of zebrafish.

It is to be noted that T-MPO larvae eventually die at around 10 dpf (data not shown). It is conceivable that the absence of the swim bladder, which compromises the navigation, in conjunction with the defective development of the digestive tract negatively impact on feeding and nutrients absorption, which may concur to premature death. S-MPO larvae also showed a reduced survival rate (10% less compared to controls), further indicating that the amount of available CD in the early stage of development may be a limiting factor for zebrafish survival.

Zebrafish embryo development fully depends on yolk in the first 3–4 days [35], [39]. Vitellogenin is the main precursor of yolk proteins in eggs of oviparous animals [50]. Accumulating evidence indicate that the ovarian hydrolase responsible for the conversion of vitellogenin into yolk proteins is actually the lysosomal protease CD. Both CD and vitellogenin co-exist within endosomes of rainbow trout oocytes [51]. CD has been shown to process vitellogenin and yolk proteins during oocyte growth and embryo growth in birds [52], *Xenopus laevis* [53], seabream fish [54] and in salmonids [55]. Therefore, it is likely that CD accomplishes a similar function in the processing of yolk proteins also in zebrafish. This hypothesis is supported by the fact that in S-MPO zebrafish, in which a small amount of CD arising from maternal mRNA translation was present, the yolk was normally processed and consumed as in controls. Based on developmental milestones [48], the phenotype of T-MPO 4 dpf larvae appears more close to that of normal 3 dpf larvae at the protruding-mouth stage. In contrast, CTRL and S-MPO 4 dpf larvae reached the pSB+ phase (following inflation of posterior swim bladder lobe), in which the head shows more anterior mouth position [48]. We propose that the delay in the development of the entire body and of several organs (eye, digestive tract) in T-MPO zebrafish arises from the impaired utilization of yolk consequent to the lack of CD.

Another important consequence of the lack of CD observed in new born zebrafish was the altered development of the eye, that showed a reduced dimension, and of the RPE cells, which were deprived of microvilli. By 7 dpf the dimension of the eye reached the same dimension as controls (not shown). However, the integrity of the RPE layer was not investigated in these larvae. Iridophores were, apparently, not affected, indicating that the vesicles containing guanine crystals responsible for the light reflection [56] normally formed in the absence of CD. The RPE plays critical roles for the embryonic eye formation and also for maintaining visual functions in adult [57], [58]. During eye development, RPE regulates retinal lamination and PRC morphogenesis [59], [60]. Consistently, we found that CD is highly expressed in RPE cells. In adults, RPE cells secrete growth factors and neurotransmitters and allows the blood to retina transport of water, ions and nutrients [58]. Therefore, the microphtalmia in T-MPO zebrafish could arise from the reduced supply of water, ions, nutrients and growth factors normally provided by RPE cells. The RPE layer provides a protective shield to PRC [58]. In addition, phagocytosis and lysosomal degradation of outer segment debris shed by PRC is accomplished by RPE cell microvilli, which interdigitate in the PRC layer, and is essential for PRC function [58]. It has been shown that CD represents the 7% of the total hydrolases secreted by RPE cells [59]. Lysosomal enzymes secreted by the RPE cells into the interphotoreceptor matrix have been suggested to also participate in the digestion of shed outer segments and of interphotoreceptor matrix components [59]. Finally, RPE cells also perform the isomerization of all-trans retinal acid to 11-cis-retinal during the visual cycle [58]. Considering the multiple roles of RPE, it is very likely that in CD KD zebrafish visual defects occur.

CD has been shown to play important roles in eukaryotic cell functioning and tissue turnover and remodeling, being involved in protein maturation and degradation and in cell death as well as in cell proliferation [61]–[63]. On this ground, it is expected that the lack of CD altered the autophagy flux and the rate of cell death *vs* cell proliferation in several organs in T-MPO zebrafish. Investigations in this direction are underway in our laboratory.

Defective ontogenesis of the swim bladder, altered development of the eye and of the retina, defective absorption of the yolk and shortened lifespan have been reported in zebrafish carrying inactivating mutations in genes involved in the biogenesis, trafficking and fusion of endosomes, lysosomes and lysosome related organelles [35], [36], [49], [64]. The similarities of these phenotypes underscore the role of lysosomal proteolysis in development.

In conclusion, the present findings show that CD plays critical roles in tissue homeostasis and organ development in zebrafish. Given the similarity of organ development, organization and function between zebrafish and mammals [30], these data suggest that defective CD-mediated lysosomal proteolysis may contribute to several pathologies in humans also, including the blinding diseases, such as the Leber's congenital amaurosis, the Best macular dystrophy and the age-related macular degeneration, in which the RPE layer is primarily affected [65], [66]. In this respect, it is worth noting that defective CD activity in human RPE cells has been linked to age-related macular degeneration [67], [68].

## Materials and Methods

### Zebrafish husbandry

Zebrafish (*Danio rerio*) were maintained as previously described [34] and staged based on developmental time and morphological criteria according to published guidelines [39], [48]. Fish were kept under a 14 hours-light and 10 hours-dark photoperiod at approximately 28°C. Following fertilization, eggs were collected and embryos (wild type or micro-injected) were raised at 28°C under standard laboratory conditions. Unless otherwise specified, embryos were grown in the presence of 0.003% 1-phenyl-2-thiourea (PTU) to prevent formation of melanin pigment. Experimental procedures related to fish manipulation followed the recommendation reported in [69] and were conform to the Italian regulations protecting animals used for research purposes, including those of the DL 116/92.

### Reverse-Transcription Polymerase Chain Reaction

Total RNA was extracted according to the TRIzol LS reagent protocol (Invitrogen Co, Carlsbad, CA, US) from UFE, embryos at 30% epiboly (corresponding to approximately 4.66 hpf), or at 1 and 2 dpf and larvae at 3 and 4 dpf (n = 50 of each sample). Aliquots (3.5 µg) of DNAse I-treated total RNA were retro-transcribed using the RevertAiD H Minus First Strand cDNA Synthesis Kit (Fermentas, Burlington, CA, US). Multiplex RT-PCR (35 cycles) was performed according to manufacturer's instructions with DyNzyme EXT DNA Polymerase (Finnzymes OY, Espoo, Finland) starting from 2 µl of cDNA and using a final concentration of 1 µM zebrafish CD primers (forward primer: 5′-CAT ATT AGA CCG CAC AAC AAT AA; reverse primer: 5-ATC ATC ATA ATG CTA AAC TCC GT) and 0.08 µM zebrafish β-actin 1 primers (forward primer: 5′-GTA TCC ACG AGA CCA CCT TCA; reverse primer: 5′-GAG GAG GGC AAA GTG GTA AAC). These conditions avoid saturation of the PCR products and were determined in preliminary separate RT-PCR reactions for each couple of primers. Primers were from MWG-BIOTECH AG (Ebersberg, Germany). The expected multiplex RT-PCR products were a 549 base pair (bp) amplicon for zebrafish β-actin 1 mRNA and a 1380 bp amplicon for zebrafish CD mRNA. The RT-PCR products were analyzed by agarose gel electrophoresis. DNA ladder was purchased from Fermentas (O'GeneRuler DNA Ladder Mix, Fermentas).

### Quantitative Real Time RT-PCR

Total RNA was extracted as described above and the cDNA was synthesized by QuantiTect SYBR Green RT-PCR (Qiagen, Hilden, Germany) according to manufacturer's protocol. qReal Time RT-PCR experiments were performed according to QuantiFast SYBR Green PCR protocol (Qiagen). β-actin 1 was chosen as reference gene. The primers used were QT02072287 for zebrafish CD and QT02174907 for zebrafish β-actin, purchased from Qiagen. 75 ng of template was used in each reaction. For the negative control, DDH20 RNASE-DNASE free (Qiagen) was used instead of template cDNA. Two independent experiments, each run in triplicate, were performed using an Applied Biosystems Abi Prism 7000 Sequence Detection System (SDS) (Applied Biosystems, Foster City, CA, US). Amplification was done according to the cycling program of QuantiFast SYBR Green PCR (Qiagen) followed by a dissociation stage according to Abi Prism 700 SDS for the Sybr Green assay. Amplification and dissociation curves generated by the SDS 1.1 software were used for gene expression analysis. Ct values were obtained for each reaction and the average was calculated. The relative mRNA expression of each transcript was calculated according to the comparative 2-Delta-Delta-Ct method [70] and the value stood for an n-fold difference relative to the calibrator. UFE ΔCt served as calibrator (relative gene expression values for calibrator were set to 1). Data are given as average ± SD. The Student's *t* test was employed for statistical analysis. A p<0.05 value was assumed as significant.

### Full length cloning of zebrafish Cathepsin D

Zebrafish CD cDNA was generated by RT-PCR using 10 µM of primers (forward primer: CATATTAGACCGCACAACAATAA, reverse primer: ATCATCATAATGCTAAACTCCGT), subcloned into the plasmid pcDNA 3.1 Zeo (Invitrogen Co) and subjected to automated sequencing (ABI PRISM 3100, Applied Biosystems). Primers were from MWG-BIOTECHAG. The plasmid carrying the human CD cDNA has already been described [41].

### Cell cultures and plasmid transfections

Embrionic zebrafish fibroblast at 1dpf PAC2 cells were cultured under standard conditions (28°C; room atmosphere) in Leibovit's L-15 medium (Sigma-Aldrich Co, St. Luis, MO, US) supplemented with 40% of heat-inactivated fetal bovine serum (Lonza Basel, Switzerland), 20 mM penicillin, erythromycin, streptomycin solution (Sigma-Aldrich Co), 20 mM glutamax (Invitrogen Co), 50 mg Gentamicine sulfate salt (Sigma-Aldrich Co). Human neuroblastoma SH-SY5Y cells were cultured under standard conditions (37°C; 95% air:5% CO2) in 50% MEM and 50% F12 nutrient medium (Sigma-Aldrich Co), supplemented with 10% fetal bovine serum, 2 mM glutamax, and 1% of a penicillin-streptomycin solution. pcDNA3.1Zeo- plasmid transfections were performed by Lipofectamine 2000 (Invitrogen Co) following manufacturer's protocol.

### Genomic DNA extraction, cloning and sequencing

A pool of 5 larvae at 4 dpf was dissolved in 10 mM Tris-HCl pH 7.5, 1 mM EDTA, 50 mM KCl, 0.3% Tween 20, 0.3% NP_4_0 and incubated for 10 minutes at 98°C. 0.1 U Proteinase k (Roche Diagnostic, Roche Werk, Penzberg, Germany) was added to the lysis buffer and incubation continued for 2 h at 55°C. The enzyme was inactivated at 98°C for 10 min. The lysate was used as source of genomic DNA. The region of 622 bp comprehensive of the S-MPO complementary sequence was cloned from 1 µg of genomic DNA with 0,5 µM of primers (forward primer: ACGAACACTAAGTGACTCTGGCAGA; reverse primers: ATCACGTTCCACCATGTCGACACT). The amplicon was analyzed by 2.5% agarose gel electrophoresis, extracted with the DNA Extraction from agarose gel kit (Qiagen) and sequenced (ABI PRISM 3100, Applied Biosystems).

### Zebrafish cathepsin D affinity chromatography purification

4 dpf larvae (n = 500) were processed for Pst A affinity chromatography as previously reported [43]. All steps were performed on ice. Micro Bio-spin chromatography column (Bio-Rad, Hercules, CA, US) was filled with 100 µl of Pepstatinyl-agarose solution (P2032; Sigma-Aldrich Co), washed five times with the chromatography washing buffer (0.4 M NaCl, 0.1% Triton X-100, 0.1 M sodium formate, pH 3.5) alone and stored at 4°C. The samples were homogenized in 1 ml of chromatography lysis buffer (0.2% Triton X-100; 1 M NaCl; 0.1 M sodium formate buffer pH 3.5) containing protease inhibitor cocktail without Pst A (P2714; Sigma-Aldrich Co) using a tissue homogenizer. The homogenate was clarified by two centrifugations at 13.000 rpm for 10 minutes and then applied to the column for binding (2 h at 4°C with gently shacking). The column was washed ten times with 1 ml of washing buffer and the bound fractions were eluted with 3×500 µl of elution buffer (50 mM Trizma-HCl, pH 8.3). Remnant proteins bound to the column after elution were removed by directly adding Laemmli buffer 1 X into the column. The eluted fractions were pooled and salts and detergents removed by acetone precipitation. Precipitated proteins from each fraction were denatured with 100 µl of Laemmli buffer 1 X and resolved by SDS-PAGE.

### Mutagenesis and in vitro CD mRNA synthesis

Mis-match CD cDNA was generated by the 3SM mutagenesis method [71] using the following primers (the eight mutagenized nucleotides are indicated in capital letter): F-rescueCD 5′-CtC Ttg ctc gtt gct gcc ttt ttc tgc-3′; R-rescueCD 5′-Gaa Agc Aat CcG cat gtt ggc gag ccc tca gga-3′. Mutant cDNA was subjected to automatic sequencing (ABI PRISM 3100, Applied Biosystems). A corresponding mutant mRNA was produced by mMESSAGE mMACHINE SP6 kit (Ambion, Applied Biosystems).

### MPO-mediated knock-down of Cathepsin D and rescue experiments

Two 25 bp MPO were designed against, respectively, the ATG region (T-MPO) and the exon 2 - intron 2 splicing region (S-MPO) of zebrafish CD RNA. Both oligonucleotides were purchased from Gene Tools (Gene Tools, LLC, Philomath, OR, US). The MPOs were diluted in phenol red micro-injection solution. In the control injections (CTRL) the Gene Tools Standard Control morpholino oligonucleotide 25-mer was used. Preliminary experiments were performed to determine optimal concentrations for each MPO. For the experiments, 5 ng of T-MPO or 8 ng of S-MPO were micro-injected in fertilized eggs at the one/two-cell stage using a Nanoject II injection device (Drummond Scientific, Broomall, PA, US). The rescue experiment was performed by injecting the T-MPO (5 ng/egg) together with the mutant not-matching CD mRNA (200 pg/egg). CD expression was determined at 2 dpf (two independent experiments, total n = 40 for each type of injection) and at 4 dpf (three independent experiments, total n = 60 for each type of injection) by immunoblotting. The extent of the phenotype rescue was assessed at 4 dpf.

### Immunoblotting

CD expression was assessed by immunoblotting. Eggs and embryos/larvae were mechanically de-yolked in PBS/protease inhibitor cocktail (P8340; Sigma-Aldrich CO) solution and homogenized directly in Laemmli Buffer using an ultrasonic cell disruptor XL (Misonix, Farmingdale, NY, US). Bradford assay was used to measure the protein content. Homogenates (35 µg) were resolved by SDS-PAGE and thereafter blotted onto nitrocellulose sheet. Saturation was performed with PBS/3% milk/0.2% Tween-20 solution and washes were performed with PBS/0.05% Triton X-100 solution. Mouse monoclonal antibody against β-actin (Sigma-Aldrich Co) or rabbit polyclonal antibody against rat CD produced in our laboratory [43] were used as primary antibodies. Horse Radish Peroxidase-conjugated Goat anti-rabbit or anti-mouse antibodies (Bio-Rad) were used as secondary antibodies as appropriate. Bands were imaged and subjected to densitometry using the VersaDOC Imaging System (Bio-Rad) apparatus equipped with the software Quantity One (Bio-Rad).

### Histology and immunofluorescence

For histological analyses by light microscopy, 4 dpf embryos were collected and fixed overnight at 4°C in 10% neutral buffered formalin (Sigma-Aldrich Co). Fixed embryos were embedded in 1% agarose in order to help correct positioning and sectioning (transversal sectioning, rostral to caudal). The agarose blocks were then processed as described by Sabaliauskas and colleagues [72]. Briefly, the blocks were dehydrated by ethanol gradient and, after xylene diafanization, embedded in paraffin. Sections (4 µm) were cut using a manual microtome (Leica Microsystems AG, Wetzlad, Germany), mounted on glass slides (Superfrost ultra plus microscope slides, Thermo Scientific, Braunschweig, Germany), air-dried over night at RT and incubated at 70°C for 30 minutes before hematoxylin-eosin staining. Immunofluorescence on sections were carried out using mouse monoclonal antibody anti zebrafish α-actin (Sigma-Aldrich Co,) in order to reveal eye microvilli, rabbit polyclonal antibody against rat CD produced in our laboratory [43], followed by secondary antibodies, either IRIS-2 (green fluorescence)- or IRIS-3 (red fluorescence)-conjugated goat-antirabbit IgG or goat-antimouse IgG (Cyanine Technology SpA, Turin, Italy) incubation. Nuclei were revealed by 4′,6-diamidino-2-phenylindole (DAPI) staining. In negative control experiment, the primary antibody was omitted (not shown). Antibodies were diluted in PBS/0.1% Triton X-100 solution containing 5% of BSA. Observations were performed by two independent investigators with a Leica DMI 6000B fluorescence microscope (Leica Microsystems AG) equipped with the software Leica Application Suite version 1.8.0 (Leica Microsystems AG). Representative images of at least three to five independent experiments are shown.

### Whole fish imaging and statistics

To assess the external phenotype, including the measurements of body lengths and eye area, anesthetized fish were immersed in 1% methylcellulose and imaged with a Leica MZ10F Modular Stereomicroscope interfaced to a CCD camera and Leica Application Suite (version 1.8.0) software (Leica Microsystems AG). The micro-injection experiment CTRL/T-MPO/S-MPO was repeated 5 times in double (two different operators). We examined a total of 300 larvae at 4 dpf for CTRL or T-MPO or S-MPO micro-injection. At 4 dpf two representative image sets were taken for each experiment. Each image set included one CTRL, T-MPO and S-MPO larvae whose phenotype was representative of the population. Effective eyes dimension (area), larvae length (standard length, SL) and oro-anal tract length (snout-vent length, SVL) were measured by the software ImageJ (National Institute of Health) using n = 10 images. The micro-injection experiment CTRL/T-MPO/RESCUED was repeated 3 times. We examined a total of 150 larvae at 4 dpf for CTRL or T-MPO or RESCUED micro-injection. At 4 dpf representative images were taken for each experiment. The image set shown includes one CTRL, T-MPO and RESCUED larva whose phenotype was representative of the population. A *p≤0.05 or **p<0.01 according to the Student's *t*-test was considered to be statistically significant.
